# Effects of Furosemide on Cochlear Neural Activity, Central Hyperactivity and Behavioural Tinnitus after Cochlear Trauma in Guinea Pig

**DOI:** 10.1371/journal.pone.0097948

**Published:** 2014-05-16

**Authors:** Wilhelmina H. A. M. Mulders, Kristin M. Barry, Donald Robertson

**Affiliations:** The Auditory Laboratory, School of Anatomy, Physiology and Human Biology, The University of Western Australia, Crawley, Western Australia, Australia; University of Regensburg, Germany

## Abstract

Cochlear trauma causes increased spontaneous activity (hyperactivity) to develop in central auditory structures, and this has been suggested as a neural substrate for tinnitus. Using a guinea pig model we have previously demonstrated that for some time after cochlear trauma, central hyperactivity is dependent on peripheral afferent drive and only later becomes generated intrinsically within central structures. Furosemide, a loop diuretic, reduces spontaneous firing of auditory afferents. We investigated in our guinea pig model the efficacy of furosemide in reducing 1) spontaneous firing of auditory afferents, using the spectrum of neural noise (SNN) from round window recording, 2) hyperactivity in inferior colliculus, using extracellular single neuron recordings and 3) tinnitus at early time-points after cochlear trauma. Tinnitus was assessed using gap prepulse inhibition of acoustic startle (GPIAS). Intraperitoneal furosemide, but not saline, caused a marked decrease in both SNN and central hyperactivity. Intracochlear perfusion with furosemide similarly reversed central hyperactivity. In animals in which GPIAS measurements suggested the presence of tinnitus (reduced GPIAS), this could be reversed with an intraperitoneal injection with furosemide but not saline. The results are consistent with furosemide reducing central hyperactivity and behavioural signs of tinnitus by acting peripherally to decrease spontaneous firing of auditory afferents. The data support the notion that hyperactivity may be involved in the generation of tinnitus and further suggest that there may be a therapeutic window after cochlear trauma using drug treatments that target peripheral spontaneous activity.

## Introduction

A common side-effect of hearing loss is tinnitus, a phantom hearing sensation described as hissing or ringing in the ears [1]. Estimates of the prevalence of chronic tinnitus range from 10 to 15% of the adult population [2]–[5] but the incidence rises sharply in specific groups such as the elderly, workers in noisy environments and war veterans [6], [7]. In about 20% of sufferers, tinnitus significantly affects daily life [8]. A number of previous studies in humans have suggested that the loop diuretic, furosemide may reduce tinnitus in some sufferers [9]–[11]. The present paper investigates the possible physiological mechanism of such an action.

Although many human studies have described abnormal activity within auditory pathways of tinnitus sufferers [12]–[15], the exact neural substrate is unknown. Animal models of hearing loss have shown increased spontaneous firing rates in central auditory structures (hyperactivity), alterations in neural synchrony, as well as reorganization [16]–[21], but exactly how these changes contribute to the development of tinnitus is still debated.

Because primary auditory afferents do not show increased spontaneous firing rates after common types of acoustic trauma [1], [18], [22], it is often assumed that central hyperactivity is generated intrinsically and is not dependent on peripheral cochlear activity [1], . However, using a guinea pig model of acoustic trauma, we have shown that treatments that eliminate or reduce primary auditory nerve firing (cochlear ablation, cochlear cooling or cochlear perfusion with CoCl_2_), can significantly reduce hyperactivity in inferior colliculus [18], [25]. Interestingly, this reduction of hyperactivity could only be fully achieved within the first 6 weeks after trauma, but not at later recovery times [26]. Based on these findings we have hypothesized that there are two distinct stages following cochlear trauma. In the first stage, central hyperactivity is the result of hyperexcitability of central neurons and is still dependent on peripheral afferent drive. This drive comprises the spontaneous firing of surviving primary afferent neurons, which is still present despite the fact that acoustic trauma reduces sensitivity to sound. In the second stage, central neurons become so excitable that they begin to generate their own intrinsic spontaneous firing and hyperactivity therefore becomes relatively independent of peripheral afferent input [27]. If hyperactivity is involved in the development of tinnitus, this suggests there may be a therapeutic window for recent-onset tinnitus in the first stage, using treatments that reduce cochlear afferent firing.

Furosemide is known to reduce primary auditory nerve firing [28]. Therefore in the present study we investigated, in our guinea pig model of cochlear trauma, the effect of furosemide on spontaneous firing of the auditory afferents, on hyperactivity in inferior colliculus and on behavioural measures of tinnitus.

## Materials and Methods

### Ethics Statement

The experimental protocols were approved by the Animal Ethics Committee of The University of Western Australia (03/100/1007) and were carried out in accordance with the Guidelines from the National Health and Medical Research Council Australia regarding the care and use of animals for experimental procedures. All surgery was performed under anaesthesia and all efforts were made to minimize suffering.

### Animals

**Figure 1:**
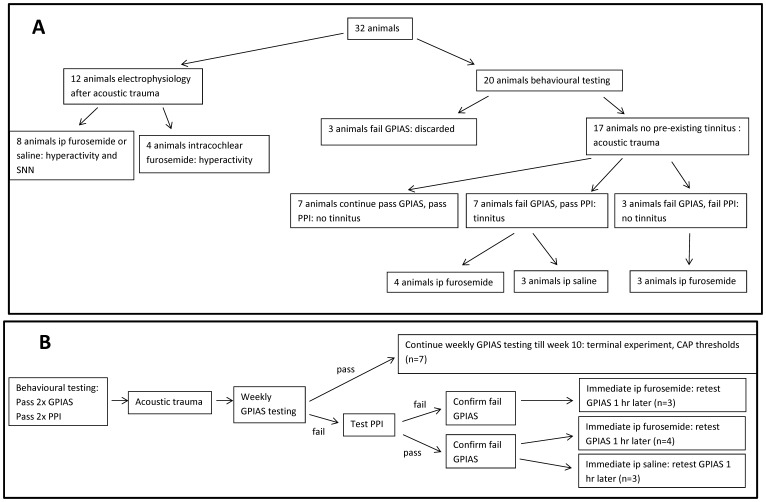
A: Overview of all animals used in the present study as allocated to the different groups. B: Schematic representation of the experimental design of behavioural experiments.

Thirty-two young adult pigmented guinea pigs of either sex were used. Animals (Tricolor strain) were derived from a local breeding colony at the University of Western Australia. Twenty of these animals were used to assess the therapeutic effects of intraperitoneal injection of furosemide (80 mg/kg) on tinnitus. The remaining twelve animals, weighing between 255 and 395 g at the time of acoustic trauma (10 kHz 124 dB SPL, 2 hours), were used to assess the effect of furosemide on spontaneous activity of the auditory nerve and on central hyperactivity measured in the central nucleus of the inferior colliculus (CNIC) 2 weeks after acoustic trauma. In eight of these animals furosemide (n = 4) or saline (n = 4) was administered intraperitoneally and in the remaining four animals furosemide was administered by intracochlear perfusion (see Fig. 1A).

### Behavioural Analysis for Tinnitus: GPIAS and PPI

Behavioural testing for tinnitus consisted of gap prepulse inhibition of acoustic startle (GPIAS) and Prepulse Inhibition (PPI). GPIAS is a variation of PPI. PPI occurs when a weaker pre-stimulus, or prepulse, inhibits the reaction to a stronger stimulus. GPIAS consists of a comparison between two conditions. Both conditions consist of a background noise and startle pulse which elicits a startle response. However, in one condition, there is a gap within the continuous background noise which precedes the startle pulse. The gap in this case works as a prepulse. In normal animals, this condition results in inhibition of the startle response. It is thought that animals experiencing tinnitus that is qualitatively similar to the background noise, show decreased startle inhibition, i.e. deficits of GPIAS, because the tinnitus “fills in” the gap in the background noise [29], [30]. Turner et al. compared GPIAS tests to other behavioural animal models of tinnitus and showed a strong positive correlation between the different methods [30].

A deficit in the GPIAS test could also be due to hearing loss (when an animal does not hear the background noise it cannot detect a gap therein) and therefore a PPI test is performed in parallel to the GPIAS test, using for the prepulse the same parameters as for the background noise in order to establish that the animal can hear the prepulse/background noise. Therefore, animals that fail GPIAS testing but pass PPI testing are thought to have tinnitus [29], [30].

Behavioural tests were performed on 20 animals. Seventeen animals, weighing between 332 and 649 g at the time of surgery, were exposed to a unilateral acoustic trauma in order to induce a hearing loss and tinnitus. The remaining 3 animals showed a GPIAS deficit before any cochlear trauma was performed and were therefore discarded from this study.

For behavioural testing, animals were mildly restrained in a clear polycarbonate holder which was placed on a custom-designed force transducer within a dark soundproof room. Just above the animal’s head, two speakers were placed, one to administer the startle stimulus (Radio Shack 401278B; 115 dB SPL, narrowband noise, centre frequency 1 kHz, bandwidth 100 Hz, 20 ms duration, 0.1 ms rise/fall time,) and the other one for continuous background noise (for GPIAS) or a prepulse sound stimulus (for PPI) (Beyer DT 48). Custom-designed software (kindly provided by R. Salvi and D. Stolzberg) and commercial hardware (Tucker-Davis technologies) was used to deliver sound stimuli and record output from the custom-designed startle platform. Acoustic stimuli were calibrated using a ½″ microphone positioned at the location of the animal’s external ear canal.

Background noise for GPIAS consisted of a narrowband noise centred at either 8 or 14 kHz (3dB bandwidths = 1 kHz). These two frequency bands were chosen to fall within the centre of peripheral hearing loss (14 kHz) and just below the region of hearing loss (8 kHz). The 14 kHz region is also the region of highest hyperactivity [31] in line with observations in human tinnitus subjects that the tinnitus pitch shows a strong correlation with the frequency region of hearing loss [32]. Intensity could be set at either 60 or 70 dB SPL. For each individual animal the lowest of these two intensity settings for which significant GPIAS (p<0.05, see below: data analysis) would occur was determined and this level was then used throughout further testing for each animal. In the PPI tests the characteristics of the prepulse for PPI were identical to the background noise characteristics for GPIAS. Animals had to pass GPIAS and PPI twice before a cochlear trauma was performed.

A behavioural test consisted of 50 presentations of the startle stimulus with varying intervals (20–30 s) between presentations. Each test consisted of either the 8 or 14 kHz background noise (GPIAS) or pre-pulse stimulus (PPI). During GPIAS testing the startle stimulus was embedded in the continuous background noise and half of the trials contained a 50 ms gap which preceded the startle stimulus by 100 ms (ISI 50 ms). The order of gap (G) and no gap (NG) trials was randomized. During PPI testing the startle stimulus was embedded in silence, and a 50 ms prepulse was presented in half of the trials, but otherwise the PPI test was identical to the GPIAS test. A noise floor test was also performed to obtain a recording of the background level of movement for each animal (20 trials, with no startle or background noise present).

Each testing session contained three behavioural tests. First each animal was allowed to habituate in the soundproof room for 5 min before commencement of testing. Then the first test (50 trials see above) was either a GPIAS or PPI test, using either 8 or 14 kHz background noise or prepulse, respectively. The second test was a noise floor test to ensure that all startle data were above the noise floor in all experiments. Then the third test was again either a GPIAS or PPI test, using the alternate frequency background noise or prepulse, respectively. The order of using 8 and 14 kHz background noise/prepulse was alternated between sessions. There was at least one day between testing sessions. Only one testing session was conducted per day for each animal, with the only exception being the day of treatment. On this day the treatment testing session was conducted 1 hour after the previous session. No animal went through more than three testing sessions in one week in order to reduce the possibility of habituation and acclimatization [29], [33], [34].

After each test it was determined whether an animal had failed or passed the GPIAS or PPI test. A Mann-Whitney statistical test was applied comparing startle amplitudes with and without GAP or pre-pulse. The animal was deemed to have failed the test when there was no significant difference between the gap/prepulse and no gap/no prepulse conditions. Passing the test was characterized by a significant difference (significance level p<0.05) between the two conditions.

After cochlear trauma, weekly GPIAS testing resumed. When an animal passed the GPIAS test the weekly testing continued. When an animal failed the GPIAS testing (i.e. no significant GPIAS), a PPI test was performed. If the animal passed the PPI test (p<0.05) then two days later another GPIAS test was performed. If the animal failed this again and was categorized as a tinnitus animal, it immediately received an i.p. injection of either furosemide (80 mg/kg) or an equivalent amount of saline and was tested again (GPIAS) 1 hour later to assess the effect of treatment on behavioural signs of tinnitus. Three of the animals failed the PPI test as well as the GPIAS test (i.e. no significant pre pulse inhibition) and they also received an immediate i.p. injection of furosemide (80 mg/kg) and were tested again for GPIAS 1 hour later. After the treatment testing, animals underwent a final experiment, at least 3 days later, during which cochlear thresholds on both sides were measured.

### Recovery Surgery for Acoustic Trauma

After a subcutaneous injection of 0.1 ml atropine sulphate (0.6 mg/ml), animals received an intraperitoneal injection of Diazepam (5 mg/kg), followed 20 minutes later by an intramuscular injection of Hypnorm (0.315 mg/ml fentanyl citrate and 10 mg/ml fluanisone; 1 ml/kg). When deep anaesthesia was obtained as determined by the absence of the foot withdrawal reflex, the area of incision was shaved and animals were placed on a heating blanket in a soundproof room and mounted in hollow ear bars. A small opening (approximately 1 mm^2^) was made in the bulla and an insulated silver wire was placed on the round window. A compound action potential (CAP) audiogram [35] for the frequency range 4–24 kHz was recorded to assess the animals’ cochlear sensitivity. All sound stimuli were presented in a closed sound system through a ½″ condenser microphone driven in reverse as a speaker (Bruel and Kjaer, type 4134). The system was calibrated using a 1/8″ condensor microphone in place of the tympanic membrane and an absolute sound calibrator (Bruel and Kjaer type 4231). Pure tone stimuli (10 ms duration, 1 ms rise/fall times) were synthesized by a computer equipped with a DIGI 96 soundcard connected to an analog/digital interface (ADI-9 DS, RME Intelligent Audio Solution). Sample rate was 96 kHz. The interface was driven by a custom-made computer program (Neurosound, MI Lloyd), which was also used to collect single neuron data during the final experiments. CAP signals were amplified (1000x), filtered (100 Hz–3 kHz bandpass) and recorded with a second data acquisition system (Powerlab 4SP, AD Instruments).

When cochlear sensitivity was within the normal range [35], animals received a unilateral acoustic trauma using the closed sound system described above in the left cochlea. For this purpose animals were exposed to a continuous loud tone for 2 h (10 kHz, 124 dB SPL), whilst still anaesthetized. The contralateral ear was blocked with plasticine. Immediately after the acoustic trauma another CAP audiogram was measured, the incision was sutured and buprenorphin (0.05 mg/kg subcutaneously) was given post-operatively as analgesic. Survival time varied between 2 (electrophysiological experiments) and 10 weeks (behavioural experiments).

### Surgery for Final Experiments

Animals received a subcutaneous injection with 0.1 ml atropine followed by an intraperitoneal injection of Nembutal (pentobarbitone sodium, 30 mg/kg) and a 0.15 ml intramuscular injection of Hypnorm. Anaesthesia was maintained with full Hypnorm doses every h and half doses of Nembutal every 2 h. When deep anaesthesia was obtained as determined by the absence of the foot withdrawal reflex, the areas of incision were shaved and animals were placed on a heating blanket in a sound proof room and artificially ventilated on carbogen (95% O_2_ and 5% CO_2_). Paralysis was induced with 0.1 ml pancuronium bromide (2 mg/ml intramuscularly). The electrocardiogram was continuously monitored and heart rate never increased over pre-paralysis levels at any stage of the experiments. After the animals were mounted in hollow ear bars, the left and right cochleae were exposed and CAP audiograms were recorded on both sides with a silver wire placed on the round window as for the recovery procedures. Animals that had been used to assess effects of furosemide on GPIAS were then immediately euthanized with an injection of 0.3 ml Lethabarb (sodium pentobarbitone 325 mg/ml; VIRBAC).

During experiments testing the effects of a single acute intraperitoneal injection of furosemide or saline on central nerve activity, the spontaneous activity of the peripheral auditory nerve fibres was also assessed using the established technique of the spectrum of the neural noise (SNN) recorded from the round window of the noise-damaged cochlea. The latter recording has a prominent peak at approximately 900 Hz which can be used as a measure of the spontaneous activity of the primary afferent fibres [36], [37]. To quantify the size of the 900 Hz peak of the SNN, the amplitude of the spectrum between 700 and 1100 Hz was averaged (10 averages/time point).

To obtain extracellular single neuron recordings in the central nucleus of the inferior colliculus (CNIC) a small craniotomy (approximately 4 mm^2^) overlying the visual cortex was performed and a glass-insulated tungsten microelectrode [38] was advanced using a stepping motor microdrive along the dorso-ventral axis through the cortex into the CNIC contralateral to the cochlea subjected previously to acoustic trauma. Electrode placement in the CNIC (about 2.5 to 3 mm ventral to the cortical surface) was indicated by the presence of strong sound-driven activity with a short latency (cluster onset latencies <6.5 ms) and a systematic progression from low to high characteristic frequencies (CF) with increasing depth. We have previously confirmed histologically that these response properties correlate with location of the electrode in the CNIC [31]. The craniotomy was covered with 5% agar in saline to improve mechanical stability. When a single neuron was isolated its CF and threshold at CF were determined audio-visually and depth from the cortical surface was recorded using methods described previously [25], [39]. In all neurons the spontaneous firing rate was measured for a period of 10 s as previously reported using an identical animal model [18], [25], [31].

### Effects of Furosemide on Central and Peripheral Nerve Activity

To assess the effect of an acute i.p. injection with furosemide (Ilium, Australia, 50 mg/ml furosemide), in 8 animals, spontaneous firing rates of CNIC neurons were collected from the frequency regions between 4 and 24 kHz before (approximately 2 hours of recording) and after an i.p. injection with furosemide (n = 4 animals) or saline (n = 4 animals). These single neuron recordings were interleaved with measurements of the SNN at regular intervals (every 5 to 15 minutes). After furosemide was injected (80 mg/kg bodyweight i.p.), SNN was monitored until a significant reduction could be observed (between 20 and 30 minutes after injection). We applied the same waiting period (30 minutes) after the saline injections. From that moment single neuron recordings resumed for another 2 hours interleaved with measurements of SNN at regular intervals (every 5 to 15 minutes).

To assess the effect of intracochlear injection of furosemide, a neuron with a high spontaneous firing rate was isolated within the region of the CNIC that showed hyperactivity. Activity from this neuron was then recorded at regular intervals before, during and after the perfusion (recordings of single neurons lasted for up to 130 minutes-see figure 1 D and E). The thresholds to CF tones of the same neurons were also recorded at regular intervals to monitor effects of furosemide on thresholds (see figure 1D and E). For the perfusions, a hole was made in the cochlear apex with the use of a hooked pick and a small hole was made in the scala tympani wall of the basal cochlear turn using a syringe needle. Using a micromanipulator the tip of a glass perfusion pipette was then carefully inserted through the hole in the scala tympani wall. The perfusion pipette contained artificial perilymph (127 mM NaCl, 5 mM KCl, 1 mM MgCl2.6H2O, 1 mM NaH2PO4.H2O, 12 mM NaHCO3, 11 mM glucose and 2 mM CaCl2 at pH 7.4) with furosemide (0.5 mM or 1 mM). Perfusion rate was 2 to 3 µl/min.

### Data Analysis

To identify statistically significant differences in spontaneous firing rates before and after intraperitoneal injection with furosemide or saline, a Kruskall-Wallis test was used as the data was not normally distributed, followed by Dunn’s multiple comparison tests. The same test was used to assess the effect of furosemide and saline on SNN. We performed statistical analysis at three time points, t = 0 (time of injection) and at t = 20 (between 15–25 min after injection) and t = 120 (between 110 and 125 min after injection). For statistical analysis of the CAP threshold data a one-way ANOVA and Bonferroni’s multiple comparison tests were used.

For analysis of each GPIAS and PPI test in each animal, a Mann-Whitney statistical test was used to compare startle amplitudes with and without gap or pre-pulse (significance level p<0.05). For analysis of group data for GPIAS, the percentage suppression in the gap (G) condition compared to the no gap (NG) condition was calculated for each test (% GPIAS = (1–(G/NG))*100). Repeated measures (RM) one-way and two-way ANOVAs were performed comparing the suppression at three different time-points (before acoustic trauma, after acoustic trauma just before drug or control treatment, and after drug or control treatment).

## Results

### Behavioural Testing Data

Figure 1 shows an overview of all animals and their allocation to the different groups described in detail below (Fig. 1A) as well as a schematic representation of the overall experimental design for the behavioural experiments (Fig. 1B).

**Figure 2:**
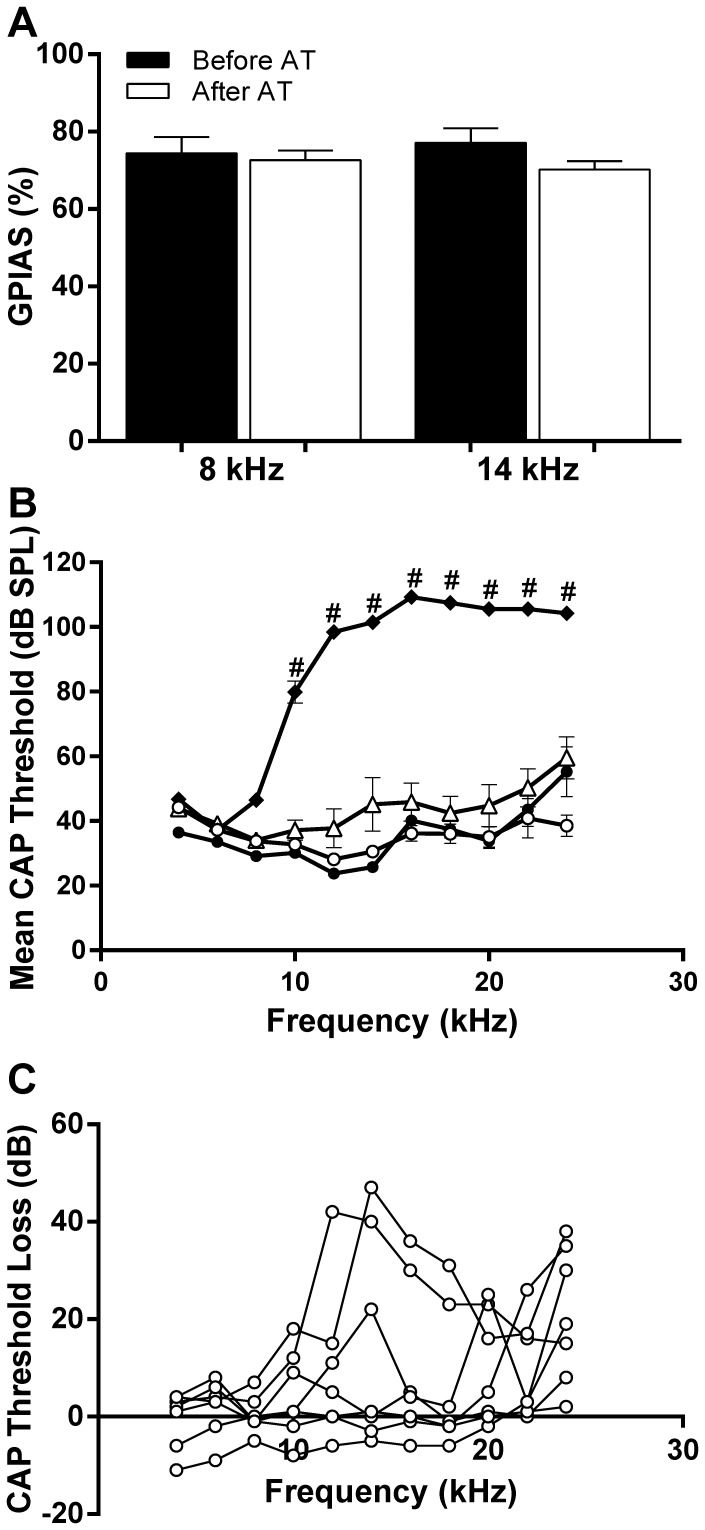
Data from 7 animals that underwent acoustic trauma and that did not develop a GPIAS deficit for a period of 10 weeks A: Histogram showing mean % GPIAS. Shown is % GPIAS measured with 8 and 14 kHz background noise before (black bars) and 10 weeks after trauma (white bars). B: CAP thresholds (mean ± SEM). Left cochlea before acoustic trauma (open circles), immediately after acoustic trauma (black diamonds) and after recovery from acoustic trauma (open triangles). Contralateral control cochlea after recovery (black circles). Statistical significance of differences between pre and post acoustic trauma data: #p<0.001. C: Threshold loss for individual animals at 10 weeks after acoustic trauma.

Of the 17 animals that showed significant initial GPIAS and PPI and then underwent acoustic trauma, 7 did not develop consistent (twice in one week) GPIAS deficits. Instead, these animals showed robust GPIAS throughout a period of 10 weeks post-acoustic trauma. Their % GPIAS is shown in figure 2A in the presence of both 8 and 14 kHz background noise before the acoustic trauma (black bars) and at 10 weeks post-acoustic trauma (white bars). The fact that these animals continued to pass GPIAS testing suggests they did not develop tinnitus throughout this 10 week period and in addition supports the notion that GPIAS is a robust and consistent phenomenon. The mean audiograms for these animals are shown in figure 2B. The mean cochlear thresholds did not show statistically significant elevations at 10 weeks post-acoustic trauma. This is most likely due to the large variations in the extent of threshold recovery between animals as illustrated in figure 2C, that shows the CAP threshold losses for each individual animal.

**Figure 3:**
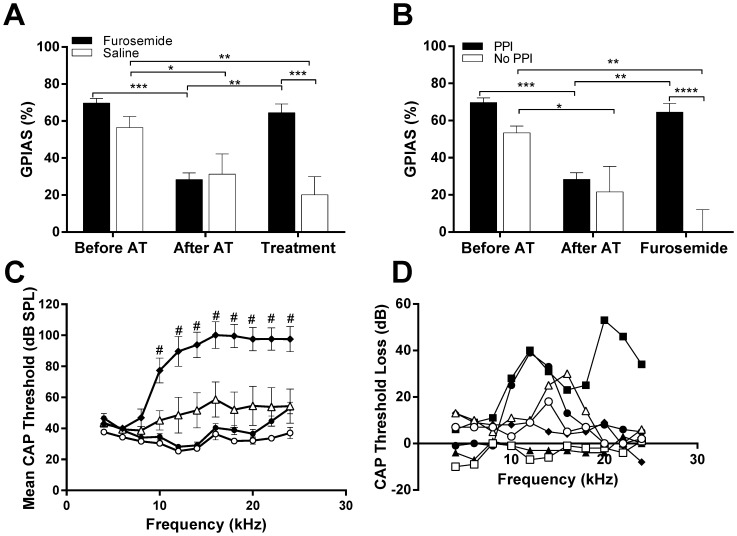
Data from animals that developed GPIAS deficits between 3 and 6 weeks post-trauma Histograms in A and B show % GPIAS before acoustic trauma (AT), after AT and after treatment. A: Data from 4 animals given furosemide (black bars) and 3 animals given saline (white bars). All animals shown in A showed significant PPI. B: Data from 4 animals with significant PPI which were given furosemide (black bars, same animals as in panel A) and 3 animals without significant PPI which were also given furosemide (white bars). Significance is shown from repeated measure two-way ANOVA and post-tests. Statistical significance: *p<0.05; **p<0.01. ***p<0.001. C: CAP thresholds from animals shown in A (mean ± SEM). Left cochlea before acoustic trauma (open circles), immediately after acoustic trauma (black diamonds) and after recovery from acoustic trauma (open triangles). Contralateral control cochlea after recovery (black circles). Statistical significance of differences between pre and post acoustic trauma data: #p<0.001. D: Threshold loss for individual animals shown in A. Black symbols are animals given furosemide and open symbols animals given saline.

Of the 17 animals that showed significant initial GPIAS and PPI and then underwent acoustic trauma, 10 animals did develop GPIAS deficits at time-points between 3 and 6 weeks after acoustic trauma. Nine of these animals developed the GPIAS deficit with 14 kHz background noise and one of them with 8 kHz background noise. They were randomly allocated to a treatment after they were separated on the basis of their PPI data. Seven of these 10 animals showed significant PPI despite the GPIAS deficit, supporting the notion that the GPIAS deficit was due to tinnitus, whereas the other 3 animals also developed a PPI deficit suggesting the GPIAS deficit may not have been due to tinnitus. Four of the 7 animals that showed evidence of tinnitus received an i.p. injection with furosemide and 3 with saline. All 4 animals that received an i.p. injection with furosemide showed individually a return of significant GPIAS, in contrast to the 3 animals that received an i.p. injection with saline. These two groups of animals with intact PPI and different treatments are shown in Figure 3A. Note that only the GPIAS outcomes for the background noise condition (i.e. 8 or 14 kHz centred noise) that revealed the deficit are shown in the group data and this will be done throughout the remainder of this paper. Statistical analysis revealed a significant interaction of time and treatment (F(2, 10) = 10.15, p<0.01). Post-hoc tests revealed that the animals which received furosemide showed significant (p<0.001) improvement in % GPIAS when compared to those animals which received saline. All other comparisons between the two groups were not significant. These findings suggest that furosemide but not saline eliminates the behavioural signs of tinnitus.

Further statistical comparison indicated that within the furosemide treatment group, the % GPIAS before acoustic trauma was significantly higher than after acoustic trauma (p<0.001). Additionally, there was significantly less % GPIAS after acoustic trauma than after treatment with furosemide (p<0.01). No other comparisons within the furosemide group were significant. Within the saline treated group, the % GPIAS before acoustic trauma was significantly higher than both after acoustic trauma (p<0.05) and after treatment with saline (p<0.01). No other comparisons within the saline group were significant. Mean CAP audiograms for these 7 animals that developed tinnitus are shown in figure 3C. Mean thresholds after recovery (varying between 4 and 7 weeks) were elevated compared to before acoustic trauma, but these changes were not statistically significant, most likely due to large inter-animal variation (CAP threshold loss for each individual animal shown in Figure 3D). In addition, there were no statistically significant differences between the threshold loss observed in tinnitus animals compared to the threshold loss observed in non-tinnitus animals.

The remaining 3 acoustic trauma animals that showed GPIAS deficits, also showed PPI deficits, which suggests that their GPIAS deficit may not have been due to tinnitus. These animals also received an i.p. injection with furosemide, but this had no effect on their GPIAS deficits as shown in figure 3B. In this figure the comparison is shown between the results from this group of animals with combined GPIAS deficit and PPI deficit and the group of animals described above (GPIAS deficit but no PPI deficit). There was a significant interaction of time and PPI performance (F(2, 10) = 10.15, p<.001). Post-hoc testing showed that after furosemide treatment, animals with impaired PPI showed significantly less % GPIAS than those with intact PPI (p<0.0001). These data show that when reduced GPIAS is associated with reduced PPI, furosemide cannot restore the suppression, unlike the situation when good PPI is still present. This shows that the beneficial action of furosemide is specific to those animals which may have tinnitus and it is therefore unlikely to be having a non-specific effect on startle circuitry *per se*.

Additional statistical comparisons between the intact PPI and impaired PPI animals (Fig. 3B), indicated that within the intact PPI group, the % GPIAS before acoustic trauma was significantly higher than after acoustic trauma (p<0.01). Additionally, there was a significantly higher % GPIAS after treatment with furosemide than after acoustic trauma (p<0.01). No other comparisons within the intact PPI group were significant. Within the PPI deficit group, the % GPIAS before acoustic trauma was greater than both after acoustic trauma (p<0.05) and after treatment with saline (p<0.01).

### Acute Effects of Furosemide on Peripheral and Central Neural Activity

**Figure 4:**
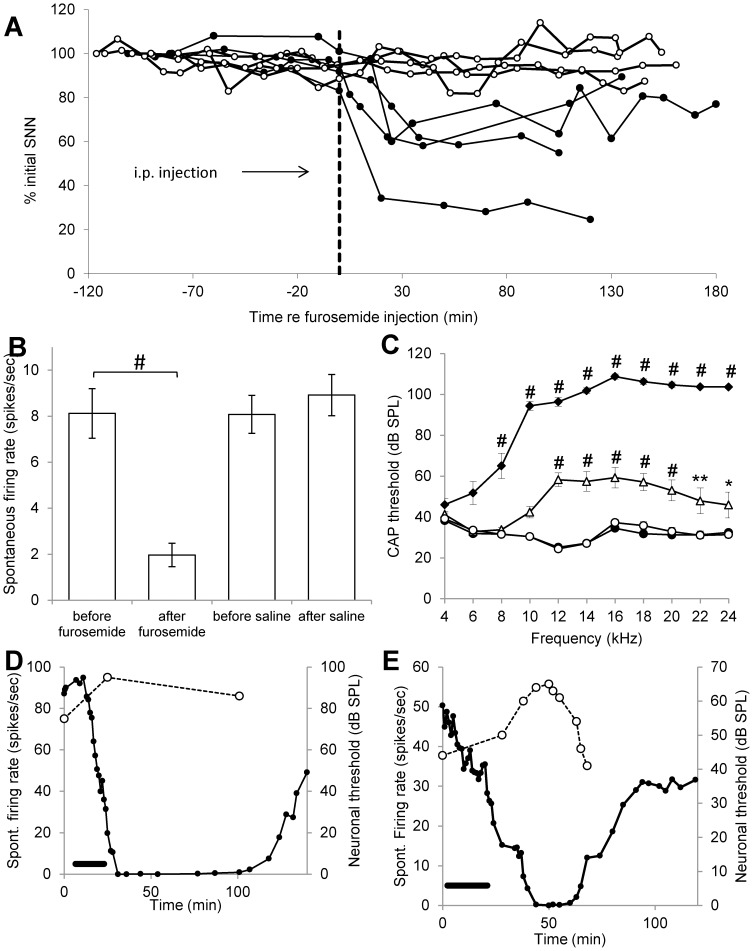
A: Spectrum of neural noise (SNN) recorded from the round window in 8 animals plotted as percentage of original value before and after an i.p. injection with furosemide (black line with filled circles n = 4) or saline (black line with open circle n = 4). Time of injection indicated by dotted line. B: Mean spontaneous firing rate of CNIC neurons recorded before and after i.p. furosemide and before and after saline (n = 4 for each group; mean ± SEM). C: CAP thresholds (mean ± SEM) at different frequencies recorded from the left cochlea before acoustic trauma (open circles), immediately after acoustic trauma (black diamonds) and after recovery from acoustic trauma (open triangles), as well as from the contralateral control cochlea after recovery (black circles). *p<0.05; **p<0.01; #p<0.001 statistical significance as compared to before trauma data. D and E: Spontaneous firing rate of 2 individual neurons from 2 different animals plotted over time before, during and after intracochlear perfusion of furosemide (black circles). Black bar indicates timing of intracochlear perfusion. Open circles indicate measurement of the neuron’s threshold to CF tones. Neuronal CF is 10 kHz and 12.4 kHz, in D and E, respectively.

Figure 4A shows the magnitude of the SNN measured from the round window in 8 different animals before and after i.p. injection with furosemide (n = 4) or saline (n = 4). The SNN can be used as a measure of spontaneous cochlear neural activity [36], [37]. In all animals injected with furosemide, the SNN showed a large reduction, which reached a maximum (40 to 70% reduction) 20 to 30 minutes after injection. The SNN remained decreased up to 3 h after injection (the maximum period recorded) in 3 animals but showed recovery to 89% of its initial value in one animal after approximately 2 hours. In contrast, in animals injected with saline, the SNN remained stable. Statistical analysis showed no significant difference between the SNN in the furosemide group and the saline group at time of injection but a significant reduction of SNN after furosemide injection compared to saline injection at t = 20 min and t = 120 min (p<0.05). These data show that i.p. administration of furosemide can decrease the spontaneous firing of the primary auditory nerve fibres.

The effects of i.p. injection of furosemide or saline on spontaneous firing rates of single neurons in CNIC, are illustrated in figure 4B. These data were obtained from 8 animals that were exposed to acoustic trauma 2 weeks previously and had therefore developed hyperactivity in CNIC (note that these animals were the same as used for the measurements of SNN described above). The mean spontaneous activity of sampled CNIC neurons before furosemide injection was 8.12±1.1 spikes/sec (ranging from 0 to 74.4 spikes/sec; n = 172 neurons from 4 animals), very similar to the mean spontaneous activity measured in 4 other animals before an injection with saline (8.1±0.8 spikes/sec, ranging from 0 to 75.9 spikes/sec; n = 235 neurons), indicating levels of neural hyperactivity in good agreement with those previously reported using identical methods [18], [26]. After the injection with furosemide (and after a decrease was observed in the averaged SNN as shown in Fig. 4A) the mean spontaneous firing rates in CNIC decreased to 1.97±0.5 spikes/sec (ranging from 0 to 44.8 spikes/sec; n = 179 neurons from 4 animals). This decrease in the spontaneous firing rates after furosemide was statistically significant, both when comparing within each individual animal as well as when using the pooled group data (all p<0.001). In contrast, an injection with saline in the other 4 animals had no effect (8.9±0.9 spikes/sec (ranging from 0 to 77.2 spikes/sec; n = 225 neurons). These data show that an acute i.p. injection with furosemide simultaneously reduced peripheral afferent spontaneous firing (SNN measurements) and lowered the hyperactive spontaneous firing rates seen in IC neurons two weeks after recovery from acoustic trauma.

We were able to record the effect of intracochlear perfusion of furosemide on 6 individual CNIC neurons that showed high spontaneous firing rates varying between 25 to 90 spikes/sec 2 weeks after acoustic trauma. In 5 of these neurons the spontaneous firing rate reduced to 0 spikes/sec and in the remaining neuron spontaneous firing rate reduced by 68.6% (from 34.7 to 10.9 spikes/sec). Four of the neurons were recorded from for long enough to observe partial recovery of spontaneous firing rate. The effect of intracochlear perfusion with furosemide on two of these neurons is illustrated in figures 4D and 4E. In addition, in these figures the thresholds of these neurons to acoustic stimulation are shown (white open circles). These show that furosemide causes a relatively small increase in neuronal thresholds and that these seem to recover at a faster rate than the spontaneous firing rate (note that thresholds for the two neurons shown in Fig. 4D,E are rather high because these neurons emanate from cochlear regions that were subjected to acoustic trauma 2 weeks before recording). These data show that an intracochlear effect of furosemide is sufficient to cause suppression of the hyperactive spontaneous firing rates seen in IC neurons two weeks after recovery from acoustic trauma.

Figure 4C shows the cochlear CAP thresholds after recovery from acoustic trauma in the 8 animals used to describe the i.p. and intracochlear effects of furosemide on neural activity. The effects of acoustic trauma were as described previously [18], [31], [40]. Immediately after acoustic trauma all CAP thresholds ≥8 kHz were significantly increased compared to before acoustic trauma values, but after 14 to 25 days (our recovery periods) thresholds recovered substantially and were only significantly different from pre-acoustic trauma values at frequencies ≥12 kHz (one-way ANOVA F(32,231) = 89.17, p<0.0001, details from post-hoc analysis shown in figure). No difference was observed between the noise-damaged (before trauma values) and contralateral control cochlear thresholds (measured after recovery from trauma), demonstrating that contralateral control cochlear thresholds remained unaffected by the cochlear trauma on the other side.

## Discussion

This paper provides for the first time, direct evidence in a single animal model that a drug treatment that reduces spontaneous firing rates in the auditory nerve, eliminates the hyperactivity in the CNIC caused by acoustic trauma, and also eliminates the behavioural signs of tinnitus.

In agreement with previous studies, acute systemic furosemide caused a reduction of spontaneous firing rates of auditory nerve fibres [28], as demonstrated by decreased SNN [36], [37]. This is most likely due to a decreased endocochlear potential, caused by an effect of furosemide on ion transporters in the stria vascularis and subsequent reduction of spontaneous neurotransmitter release from inner hair cells [28], [41]. Also in agreement with previous descriptions of the peripheral action of furosemide, the effects on spontaneous cochlear afferent activity were accompanied by relatively minor changes in cochlear neural thresholds that recovered faster than the cochlear spontaneous firing rates. Sewell [28] showed in cats, that primary afferent spontaneous firing is more sensitive than thresholds to falls in endocochlear potential induced by furosemide. This suggests that furosemide, or related drugs, could be used to selectively reduce primary afferent spontaneous firing without major effects on hearing sensitivity, which could be beneficial for any future therapeutic application.

A major finding was that acute administration of furosemide also caused a marked reduction in the spontaneous hyperactivity that developed in the CNIC after acoustic trauma. These findings are in agreement with our previous studies showing an elimination of central hyperactivity after other treatments directly reducing cochlear neural output [18], [25].

Finally, in animals with unequivocal behavioural evidence of tinnitus (reduced GPIAS and intact PPI), an i.p. injection with furosemide, but not saline, dramatically altered the result of GPIAS testing, showing a return of significant GPIAS, suggesting that furosemide reduced the level of tinnitus in these animals. The effect was robust in that all animals in this group showed a return of GPIAS after furosemide, whereas none did so after saline. Similar observations of the effectiveness of furosemide were made in 3 other animals in our laboratory that developed failures in GPIAS after mechanical lesion of the cochlea or that had pre-existing GPIAS deficits before a trauma to the cochlea (data not shown). We have previously shown that mechanical lesions also results in hyperactivity in IC neurons [42]. A specific therapeutic effect of furosemide on tinnitus is further supported by the fact that 3 animals that showed a GPIAS deficit but also a PPI deficit, did not respond positively to furosemide administration, since in these animals the GPIAS deficit was most likely due to factors other than tinnitus (see below). This suggests that furosemide does not exert its effects via a general effect on acoustic startle.

Approximately 60% of animals subjected to acoustic trauma (10 out of 17 animals) developed repeatable deficits of GPIAS. A deficit of GPIAS can be an indication of tinnitus as GPIAS testing strongly correlates with other behavioural testing paradigms for tinnitus [30]. However, a deficit in GPIAS could also be a result of hearing loss, habituation or unknown startle circuitry deficits [29], [30], [43] and we included the PPI test in our experiments in order to screen for these possibilities. Indeed, when also tested for PPI, 30% of these animals with GPIAS deficits (17% of total), also showed a PPI deficit. The remaining animals with GPIAS deficits showed significant PPI and these latter animals (41% of total numbers receiving acoustic trauma) could therefore be categorized as fulfilling criteria consistent with their experiencing tinnitus. Because the pre-pulse stimulus had the same acoustic characteristics as the background noise used in the GPIAS test, this provided valuable confirmation that the animals could still detect the background noise and that their startle circuitry was functioning normally. The percentage of animals showing tinnitus shows large variations between studies, i.e. in guinea pigs (57%; 4 out of 7 animals) [29], in mice (50%) [44] or in rats (between 30 and 75%) [45]–[48]. This could be due to species differences or to the different parameters of cochlear trauma between different studies. The fact that not all animals develop signs of tinnitus is in agreement with human population data showing that not all individuals with a hearing loss develop tinnitus [2], [4].

Taken together, our results seem to provide a mechanism for the possible therapeutic effect of furosemide in treating tinnitus, which has in fact been reported in human patients [9], [11], [49]. However, there are several important qualifications that must be placed on this conclusion.

First, the dose of furosemide (80 mg/kg) that we used for i.p injection is more than 10 times the usual hourly intravenous dose recommended in humans for treatment of severe renal and cardiovascular conditions, and it is higher than the oral dose used in the past for treatment of human tinnitus. However, direct comparison between human and guinea pig is not possible (for example different metabolic rates [50] might mean a lower dose could be used in human to produce therapeutic effects on tinnitus) and clearly more work is needed to elucidate whether lower doses that are suitable for human application can produce the physiological and behavioural effects we report here.

Second, the present study used only a single dose of furosemide and involved a single session of behavioural testing immediately after. Hence it is not known if the behavioural effects seen are long lasting or whether chronic administration of furosemide would be required to permanently suppress central hyperactivity and tinnitus.

Third, furosemide might affect CNIC hyperactivity via direct central actions, since the Na-K-2Cl co-transporter found in the inner ear is also expressed centrally [51] and a central effect cannot be ruled out by the present experiments. However, the fact that we saw changes in peripheral neural activity after i.p furosemide and also observed complete elimination of spontaneous hyperactivity in CNIC neurons after local intracochlear injection, is compatible with the notion that an intracochlear action of furosemide is sufficient to induce the central effects.

Fourth, we have only investigated the effects of furosemide between 3–8 weeks after cochlear trauma. Our previous work showed that the peripheral dependence of central hyperactivity is temporary [18], and that the central-intrinsic phase emerges at around 8 to 12 weeks post–trauma [27]. At this latter stage, both hyperactivity and tinnitus should become resistant to furosemide treatment and further animal studies are needed to test this hypothesis. Nonetheless, our present data could provide a physiological explanation for the partial success of furosemide treatment in human tinnitus patients [9], [11], [49]. Risey et al. [11] reported that 50% of patients experienced alleviation of tinnitus after furosemide administration and we suggest that patients who did not respond to furosemide, had tinnitus that was in the second, central-intrinsic, phase. Cesarani et al. [9] showed acute positive effects of furosemide in 74% of patients, with increased effectiveness when treatment was given during the first 3 months after tinnitus onset. Others also showed greater beneficial effects of furosemide treatment in patients with fresh cases of deafness than old cases of deafness, again supporting our hypothesis [10].

Finally, although the major finding of this paper strengthens the notion of a link between central neural hyperactivity and tinnitus, this relationship is by no means clear. Hyperactivity in CNIC develops rapidly (within hours after cochlear trauma) [52], as does auditory cortex hyperactivity [19], [53], but tinnitus in our animals was only observed a minimum of 3 weeks after cochlear trauma. This suggests that tinnitus development requires the involvement of other brain regions. Other brain regions that have been suggested to be involved include the limbic system and recently, the paraflocculus of the cerebellum [54], [55].
